# Acknowledgement to Reviewers of *Sports* in 2018

**DOI:** 10.3390/sports7010017

**Published:** 2019-01-09

**Authors:** Sports Editorial Office

**Affiliations:** MDPI, St. Alban-Anlage 66, 4052 Basel, Switzerland

Rigorous peer-review is the corner-stone of high-quality academic publishing. The editorial team greatly appreciates the reviewers who contributed their knowledge and expertise to the journal’s editorial process over the past 12 months. In 2018, a total of 185 papers were published in *Sports*, with a median time to first decision of 15 days and a median time to publication of 37 days. The editors would like to express their sincere gratitude to the following reviewers for their cooperation and dedication in 2018:

Abouhossein, AlirezaLavender, AndrewAerenhouts, DirkLee, ElaineAgrawal, PriyankaLee, KyuwanAlexander, MathewLi, RuiAlvares, ThiagoLinthorne, Nicholas P.Andersen, Catherine J.Lochbaum, MarcAndreoni, GiuseppeLombardi, AngelaArdigo, LucaLorenzetti, SilvioAyllon, Fernando NaclerioLouis, JulienAziz, Abdul RashidLow, DanielBadau, DanaLu, Louise WeiweiBailey, ChrisLudwig, OliverBaker, Brent A.Lugo, Ricardo GregorioBaker, JulienLupo, CorradoBallmann, ChristopherLystad, Reidar P.Barney, DavidMaltais, MathieuBeckham, GeorgeMaly, TomasBergdahl, Celena SchedeMalykh, Sergey B.Best, RussMamen, AsgeirBonaiuto, VincenzoMann, BryanBotonis, PetrosMartin, EricBoyer, BillMartini, DougBrazo-Sayavera, JavierMascherini, GabrieleBrzeziński, MichałMastorci, FrancescaBuckner, SamuelMcGuigan, MikeCabri, JanMeinke, PeterCamic, Clayton L.Mengarelli, AlessandroCamporesi, SilviaMercer, John A.Carl, DanielMethenitis, SpyridonCarroll, Kevin M.Michnik, RobertCarson, FraserMielgo-Ayudo, JuanCasal, Claudio A.Moir, Gavin L.Cholewa, JasonMorouço, PedroChtourou, HamdiNachbauer, WernerClark, Cain C.T.Nagahara, RyuClarke, NeilNaik, Ganesh R.Clemente, FilipeNickerson, BrettCollings, PaulNicolas, GuillaumeComfort, PaulNikolaidis, Pantelis TheodorosCornish, StephenNoble, James M.Coyle, Edward F.Ocampo, DiegoCramb, RobertOchiai, HirokoCrane, Barbara A.Ogueta-Alday, AnaCrawford, DerekOlek, RobertCrewther, BlairOliveira, César ChavesCrowther, RobertOlson, Michael W.Cuberos, Ramón ChacónOrtega, Félix ZuritaCumming, SeanParnianpour, MohamadDabbs, NicolePereira, AnaDawes, JayPertegal-Felices, María LuisaDe Paepe, BoelPiqueras, Pedro GómezDekker, MarloesPraça, GibsonDeli, Chariklia K.Prieske, OlafDesai, AbhishekRaizel, RaquelDiaconu-Gherasim, Loredana R.Ravuri, SudheerDing, XiaorongRaya-González, JavierDomínguez, RaulRoberson, Donald N.Drum, ScottRoberts, Claire-MarieDuncan, MikeRomann, MichaelEma, RyoichiRuiz-Barquín, RobertoEmeljanovas, ArunasRumney, PeterFavero, Terence G.Saavedra, Jose M.Fernandes, John F.T.Saavedra, MiguelFink, Rainer H.A.Sæther, Stig ArveFlack, Kyle D.Sainani, KristinFlora, ColledgeSánchez, Guillermo F. LópezFragkos, KonstantinosSantos, LuisFrederick, DiMennaSanz-Rivas, DavidFulford, JonathanSarmento, HugoGajewski, JanSatchidanand, NikhilGarcía-García, OscarSchnell, FrédéricGarcia-Ramos, AmadorSchubert, MattGehlert, SebastianSchuermans, JokeGeisler, CorinnaSchulze, GundulaGentles, JeremySekulic, DamirGeorgian, BadicuSenchina, DavidGerdin, GöranShadgan, BabakGipson, ChristinaSherman, Ryne A.Gómez-López, ManuelSiceloff, BeckyGómez-Piqueras, PedroSimović, SlobodanGrygorowicz, MonikaSinclair, Peter JamesHackett, DanielSmeuninx, BenoitHall, Eric E.Smilios, IliasHelms, Eric R.Smith, Johneric W.Harding, ChrisSole, ChristopherHarry, John R.Spang, ChristophHart, AlgerianStastny, PetrHassan, AmrStefani, LauraHassan, Amr AbdulfattahStorey, AdamHayward, David R.Suárez-Varela, María M. MoralesHealy, RobinSuchomel, TimothyHeazlewood, IanSuh, Young SooHerda, Ashley A.Suzuki, KatsuhikoHildreth, BlakeTaleb, NadineHojman, PernilleTessitore, AntonioHokanson, James F.Tiidus, PeterHolmstrup, MichaelTomczak, AndrzejHoriuchi, MasahiroTorres-Unda, JonHornsby, W. GuyToubekis, ArgyrisHosick, PeterTraustadόttir, TinnaHurst, ChristopherTravassos, BrunoIglesias-Soler, EliseoTriplett, TravisIngber, LesterTrudel, PierreIshihara, ToruTrue, LarissaIturricastillo, AitorVan Der Ham, InekeJaeger, RalfVentura, JessicaJones, MargaretVescovi, Jason D.Jozwiak, MathieuVicedo, Juan Carlos PastorJurkojć, JacekVickers, BradKagawa, MasaharuVisser, BartKam, LynnWagle, John P.Kawamura, TakujiWaller, MichaelKawczynski, AdamWarner, MartinKennedy, MichaelWatt, PeterKerhervé, HugoWax, B.Khoury, Dalia EIWiggs, MichaelKim, Hyun-DuckWilk, MichałKim, Kil-SooWillems, MarkKindy, MarkWilliams, TylerKingsley, J. DerekWilson, PatrickKirk, BenWinchester, LeeKlein, MarkusWojcik, Janet R.Klika, RiggsWoodfield, LorayneKnechtle, BeatWu, BoKnell, GregoryYoung, WarrenKonarski, JanZając, AdamKraus, KorneliusZekanowski, CezaryKump, DavidZelei, AmbrusLake, JasonZemková, ErikaLane, MichaelZubiaur, MartaLangford, T. DianneZuckerman, Scott

